# Supplementary material for: Optimal minimal residual disease threshold in pediatric acute myeloid leukemia: A retrospective cohort study based on the TARGET database
Source: PLoS Med. 2026 May 8;23(5):e1005088. doi: 10.1371/journal.pmed.1005088 (PMC13155632; doi:10.1371/journal.pmed.1005088)
Supplement: S1 Code — (ZIP) [file pmed.1005088.s002.zip › S2 code/PROJ8_26_tbl/PROJ8_26_tbl_seg2_2.pdf]

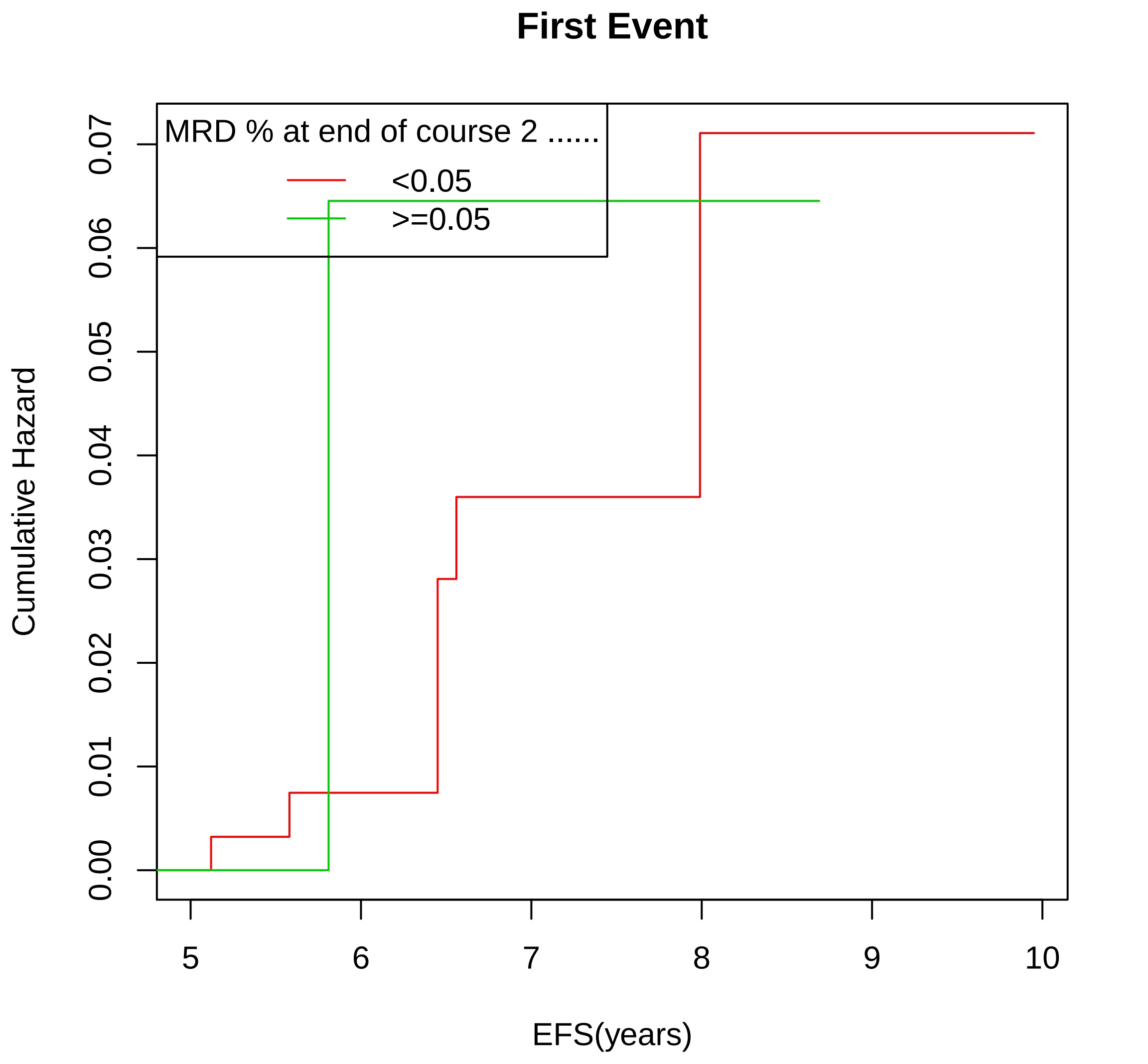
First Event
MRD % at end of course 2 ......
<0.05
>=0.05
Cumulative Hazard
0.00
0.01
0.02
0.03
0.04
0.05
0.06
0.07
5
6
7
8
9
10
EFS(years)
